# The life cycle of *Trypanosoma (Nannomonas) congolense* in the tsetse fly

**DOI:** 10.1186/1756-3305-5-109

**Published:** 2012-06-27

**Authors:** Lori Peacock, Vanessa Ferris, Mick Bailey, Wendy Gibson

**Affiliations:** 1 School of Biological Sciences University of Bristol, Bristol, BS8 1UG, UK; 2 Department of Clinical Veterinary Science, University of Bristol, Langford, Bristol, BS40 7DU, UK; 3 Royal Veterinary College, Hawkshead Lane, North Mymms, Hatfield, Hertfordshire, AL9 7TA, UK

## Abstract

**Background:**

The tsetse-transmitted African trypanosomes cause diseases of importance to the health of both humans and livestock. The life cycles of these trypanosomes in the fly were described in the last century, but comparatively few details are available for *Trypanosoma (Nannomonas) congolense*, despite the fact that it is probably the most prevalent and widespread pathogenic species for livestock in tropical Africa. When the fly takes up bloodstream form trypanosomes, the initial establishment of midgut infection and invasion of the proventriculus is much the same in *T. congolense* and *T. brucei*. However, the developmental pathways subsequently diverge, with production of infective metacyclics in the proboscis for *T. congolense* and in the salivary glands for *T. brucei*. Whereas events during migration from the proventriculus are understood for *T. brucei*, knowledge of the corresponding developmental pathway in *T. congolense* is rudimentary. The recent publication of the genome sequence makes it timely to re-investigate the life cycle of *T. congolense*.

**Methods:**

Experimental tsetse flies were fed an initial bloodmeal containing *T. congolense* strain 1/148 and dissected 2 to 78 days later. Trypanosomes recovered from the midgut, proventriculus, proboscis and cibarium were fixed and stained for digital image analysis. Trypanosomes contained in spit samples from individually caged flies were analysed similarly. Mensural data from individual trypanosomes were subjected to principal components analysis.

**Results:**

Flies were more susceptible to infection with *T. congolense* than *T. brucei*; a high proportion of flies infected with *T. congolense* established a midgut and subsequent proboscis infection, whereas many *T. brucei* infections were lost in the migration from foregut to salivary glands. In *T. congolense*, trypomastigotes ceased division in the proventriculus and became uniform in size. The trypanosomes retained trypomastigote morphology during migration via the foregut to the mouthparts and we confirmed that the trypomastigote-epimastigote transition occurred in the proboscis. We found no equivalent to the asymmetric division stage in *T. brucei* that mediates transition of proventricular trypomastigotes to epimastigotes. In *T. congolense* extremely long epimastigotes with remarkably elongated posterior ends were observed in both the proboscis and cibarium; no difference was found in the developmental stages in these two organs. Dividing trypomastigotes and epimastigotes were recovered from the proboscis, some of which were in transition from trypomastigote to epimastigote and *vice versa*. It remains uncertain whether these morphological transitions are mediated by cell division, since we also found non-dividing cells with a variously positioned, juxta-nuclear kinetoplast.

**Conclusions:**

We have presented a detailed description of the life cycle of *T. congolense* in its tsetse fly vector. During development in the fly *T. congolense* shares a common migratory pathway with its close relative *T. brucei*, culminating in the production of small metacyclic trypanosomes that can be inoculated with the saliva. Despite this outward similarity in life cycle, the transitional developmental stages in the foregut and mouthparts are remarkably different in the two trypanosome species.

## Background

Trypanosomes transmitted by tsetse in Africa cause diseases of importance to the health of both humans and livestock. Of the livestock trypanosomes, *Trypanosoma (Nannomonas) congolense* is probably the most prevalent and widespread pathogenic trypanosome in tropical Africa, being found in ruminants, pigs, dogs and other domestic animals throughout the tsetse belt [1]. In the mammalian bloodstream *T. congolense* is a small trypanosome, shorter in length than *T. brucei* and without a conspicuous undulating membrane. In the tsetse fly vector, both species develop and multiply in the midgut initially, before onward migration to the mouthparts; infective metacyclics develop in the proboscis for *T. congolense* and in the salivary glands for *T. brucei*. This difference in vector developmental site led to the subgeneric classification in current use for the African tsetse-transmitted trypanosomes: trypanosomes that develop in the midgut and proboscis are in subgenus *Nannomonas*, while those that use the midgut and salivary glands are in subgenus *Trypanozoon*[2]. In phylogenetic analyses of trypanosome species, these two subgenera appear as sister groups within the clade of African tsetse-transmitted salivarian trypanosomes [3,4].

The life cycles of *T. congolense* and *T. brucei* were described in detail in the last century (reviewed by [2]). For *T. brucei*, our understanding of the developmental cycle in the fly has been steadily augmented by information emerging from molecular and cell biology analyses: e.g. differentiation of bloodstream to procyclic forms [5,6]; major surface glycoproteins of developmental stages [7-10]; cell cycle control [11]; genetic exchange and meiosis [12,13]. Much less is known about the life cycle of *T. congolense*, but evidence to date suggests that many aspects of the developmental pathway in tsetse are similar to that of its close relative, *T. brucei*. In both species bloodstream forms differentiate to procyclics in the midgut and lose the variant surface glycoprotein (VSG) coat, but the major surface molecules of *T. congolense* are carbohydrates rather than glycoproteins as in *T. brucei*[14,15]. From the midgut both species migrate anteriorly to reach the mouthparts, using the proventriculus or cardia (the valve separating the midgut from the foregut) as a staging post. Here *T. brucei* undergoes an asymmetric division that yields one short and one long epimastigote, but this dividing stage has not been described in *T. congolense.* The short epimastigote is crucial for *T. brucei*, as it goes on to invade and colonise the salivary glands [11,16,17]. The equivalent stage that founds the proboscis infection in *T. congolense* is reported to be a trypomastigote and transformation of trypomastigotes to epimastigotes occurs after attachment in the labrum of the proboscis [2]. In addition, attached trypanosomes are found in the adjacent cibarium [18], but it is not clear if these are an extension of the proboscis population or a separate stage of development. In both species the attached epimastigotes proliferate and subsequently differentiate into infective metacyclics that are preadapted for life in the mammalian host by their protective VSG coat [19,20]. In *T. brucei* metacyclics are produced in the salivary glands, while in *T. congolense* they develop in the labrum and hypopharynx [21,22].

While attempts to produce metacyclic *T. brucei in vitro* have met limited success [23,24], the developmental cycle of *T. congolense* can be reliably reproduced *in vitro* and cultures yield large numbers of trypanosomes of different life cycle stages [25-28]. The crucial difference lies in the ability of *T. congolense* epimastigotes to attach to a plastic surface, proliferate and subsequently differentiate into metacyclics, just as they do *in vivo*. *T. brucei* epimastigotes probably need live cells for attachment, because *in vivo* there is intimate contact between outgrowths of the flagellar membrane with cells of the tsetse salivary gland epithelium [19]. EM studies show that attachment of *T. congolense* epimastigotes is via hemidesmosomes both *in vitro* and *in vivo*[20,22,29]. Comparison of shaken and unshaken cultures showed that attachment is not necessary for epimastigote division but is a prerequisite for differentiation into metacyclics [30]. The question whether the *in vitro* produced life cycle stages of *T. congolense* represent those produced in the fly has been addressed by comparison of morphology at the light and ultrastructural levels, and immunocytological analysis of the VSG coats of metacyclics [20,26,31]. In addition, the expected stage-specific cell surface markers were expressed by each of three life cycle stages (procyclics, epimastigotes and metacyclics) cultured *in vitro*[32].

The recent publication of the genome sequence of *T. congolense*[33], together with interest in using it as a convenient *in vitro* proxy to access the complete developmental cycle of the model trypanosome, *T. brucei*[32], prompts re-investigation of the *T. congolense* life cycle. This has been dealt with rather cursorily in the literature, probably because of its similarity to that of *T. brucei*, and details of transitional forms are sparse [21,34,35]. A key question is whether there is a form equivalent to the asymmetric divider of *T. brucei*[11,16,17] that has been overlooked. Here we have examined a detailed timecourse of the development of *T. congolense* in the tsetse fly to provide a comprehensive and illustrated reference to the stages in its life cycle.

## Methods

### Tsetse flies & trypanosomes

Experimental tsetse flies were from the Bristol laboratory colony of *Glossina morsitans morsitans* originally from Zimbabwe. Flies were kept at 25°C and 70% relative humidity, and fed on sterile defibrinated horse blood supplemented with 2.5% w/v bovine serum albumen (Sigma A4503) [36] and 1 mM dATP [37] via a silicone membrane. Male and female flies were used for experiments, being given the infective bloodmeal for their first feed 24–48 hours post-eclosion. The infective bloodmeal contained approximately 8 x 10^6^ trypanosomes ml^-1^ in sterile horse blood supplemented with either 60 mM N-acetyl-glucosamine (NAG) [38] or 10 mM L-glutathione [39] to increase infection rates. The infective bloodmeal for flies dissected at 2–3 days was made with sterile horse serum to aid the visualisation of trypanosomes. For examination of trypanosomes extruded in spit samples (a mixture of saliva and regurgitated foregut contents), flies were caged individually; for other experiments, flies were caged in groups of 15–25. Bloodstream form trypanosomes of *T*. *congolense* savannah 1/148 (MBOI/NG/60/1-148) [40] were grown in mice and used to infect flies. *T. b. brucei* J10 (MCRO/ZM/73/J10 [clone 1]) was used for comparison.

### Fly dissection

Flies were dissected 2 to 78 days after infection. Alimentary tracts, from the proventriculus to the rectum, were dissected in a drop of phosphate buffered saline (PBS) and viewed as wet mounts under phase contrast (x100 magnification) to search for trypanosomes. Proventriculi were removed from infected midguts and viewed separately. Proboscides from flies with midgut infection were dissected into a separate drop of PBS and teased apart, gently rubbing a fine needle down the length of the proboscis to dislodge the trypanosomes. Cibaria from flies with midgut infection were dissected into a separate drop of PBS. Trypanosomes were fixed in 2% w/v paraformaldehyde in PBS for 15 minutes in a humid chamber, followed by three PBS washes. After brief drying, preparations were stained using 4’,6-diamidino-2-phenylindole (DAPI) in Vectashield (Vector labs) mounting medium and viewed within 1 hour. Bloodstream form trypanosomes obtained from a mouse infected with *T. congolense* 1/148 (3 day infection) were fixed and stained in the same way.

### Spit samples

Spit samples were obtained from individually caged flies essentially as described by [41]. Flies were starved for approximately 48 hours before being allowed to probe onto an alcohol-cleaned microscope slide on a heating plate held at approximately 37°C; flies were fed once they had probed, or after a maximum of 30 minutes. Flies were probed on alternate days commencing 10 days after the infected feed. Saliva samples dried immediately on contact with the microscope slide and slides were stored in the dark at ambient temperature before examination. The samples were checked for the presence of trypanosomes under phase contrast (100x magnification); positive slides were fixed for 30 s in methanol, then stained and mounted with DAPI in Vectashield as above.

### Imaging & measurements

Images were recorded using a DMRB microscope (Leica) equipped with a Retiga Exi camera and Volocity version 4.1 software (Improvision). Each image was photographed under phase contrast and UV fluorescence at 400x magnification. Measurements were made on the digital images using Image J software (Version 1.41) (*http://rsb.info.nih.gov/ij/*). Dimensions measured were those used by [11] and shown in Additional file 1: Figure S1.

### Statistical analysis

Since many of the dimensions measured were likely to be internally correlated to some extent, the entire dataset was subjected to principal components analysis using the *princomp* procedure from the statistical package R (http://www.r-project.org/) to extract underlying latent variables. Only three uncorrelated factors were identified with eigenvalues greater than 1, accounting for 46.3 (factor 1), 26.1 (factor 2) and 13.4% of the observed variance. Extracted scores for factors 1 and 2 for each trypanosome were plotted. Loadings were extracted and the absolute values plotted (Additional file 2: Figure S2) to determine the extent to which each of the individual measurements contributed to the first two factors. See legend to Additional file 2: Figure S2 for details.

## Results

### Infection rates

A high proportion of *G. m. morsitans* infected with *T. congolense* savannah 1/148 established a midgut and subsequent proboscis infection ( 1). Flies were more susceptible to infection with this trypanosome strain than with *T. b. brucei* J10: most *T. congolense* midgut infections gave rise to detectable foregut infections revealed by trypanosome-positive spit samples, and the foregut infections in turn produced proboscis infections (Table 1). In contrast, many *T. b. brucei* infections were lost in the migration from foregut to salivary glands as only 30% of flies that produced positive spit samples had infected salivary glands at dissection (Table 1).

**Table 1:** **Trypanosome infection rates in*Glossina morsitans morsitans***

	*T. congolense* (Tc)	*T. b. brucei* (Tbb)
No. of flies dissected	50	87
No. of infected midguts	42/50 (84%)	48/87 (55%)
No. of infected proboscides (Tc) or salivary glands (Tbb)	39/50 (78%)	12/87 (14%)
Transmission index (TI)	39/42 (93%)	12/48 (25%)
No. of midguts with positive spit samples	34/42 (81%)	40/48 (83%)
No. of proboscides (Tc) or salivary glands (Tbb) with positive spit samples	34/34 (100%)	12/40 (30%)

Male flies were individually caged and infected with *Trypanosoma congolense* 1/148 or *T. b. brucei* J10; the infected feeds were supplemented with N-acetyl-glucosamine. Flies were dissected 4–11 weeks after infection.

### Midgut trypanosomes

**Figure 1:**
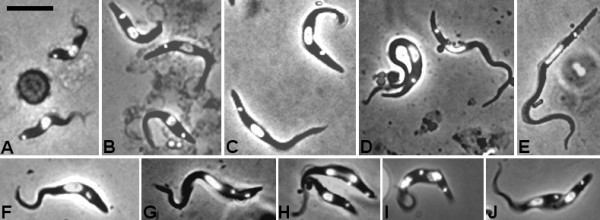
**Morphology of developmental stages of*Trypanosoma congolense.*** Fixed and DAPI-stained cells; each panel shows a merge of DAPI and brightfield images. A. Bloodstream forms. Panels B to F are procyclic trypomastigotes from the tsetse midgut 2 days (B), 6 days (C), 9 days (D) and 17 days (E) after ingestion of the infected bloodmeal. Panels F to J are trypomastigotes from the tsetse midgut in various stages of division; 2K1N (F, G), 2K2N (H-J). Bar = 10 μm.

Bloodstream form (BSF) *T. congolense* are monomorphic and mensural data show the population in infected hosts to be statistically homogeneous [42]; a pre-adapted tsetse stage analogous to the short stumpy form in *T. brucei* has not been described [2]. *T. congolense* 1/148 BSF were short, broad trypanosomes with a mean length of 17.5 μm ± 0.4 μm; the kinetoplast was near the posterior end of the cell with the nucleus occupying a central position closer to the posterior than anterior of the cell (Figure 1A; Additional file 3: Table S1). Two days after being ingested by tsetse flies, BSF had differentiated into procyclic trypomastigotes and begun to proliferate; some cells had started to elongate, with a notable increase in the distance of the kinetoplast from the cell posterior (~2 μm) and a modest increase in the kinetoplast-anterior distance (~0.6 μm); the kinetoplast was also slightly closer to the nucleus (Figure 1B; Additional file 3: Table S1). These changes reiterate those observed for differentiation of *T. brucei*[43,44].

Midgut procyclic trypanosomes gradually lengthened over time, from a mean length of 20.0 ± 0.4 μm on day 2 to 40. 9 ± 0.4 μm on day 17, i.e. doubling in length (Figures 1B to E; Additional file 3: Table S1). At the same time, the trypanosomes became more slender, halving in width from 2.6 ± 0.1 μm on day 2 to 1.3 ± 0.1 μm on day 17. Corresponding changes were seen in nuclear length and width, the nucleus becoming longer and thinner (Figures 1B to E; Additional file 3: Table S1). Keeping the kinetoplast as the point of reference, most of the increase in cell length appeared to occur at the anterior end of the cell: the kinetoplast-anterior distance increased from 17.0 ± 0.3 μm on day 2 to 35.7 ± 0.3 μm by day 17, compared to a modest increase in the kinetoplast-posterior distance of <3 μm. The relative distance between the nucleus and kinetoplast remained constant (Additional file 3: Table S1). Assuming that the increase in cell length results from posterior extension of microtubules as in *T. brucei*[44], then the apparent growth of the anterior end of the cell in *T. congolense* procyclics actually results from gradual movement of the kinetoplast and nucleus towards the posterior. It is important to note that at any one timepoint the trypanosome population was not of uniform morphology, but showed large variability both in length and shape (Additional file 3: Table S1). For example, in some trypanosomes the posterior tapered to a point, while in others the posterior was blunt.

Trypanosomes in the process of division were identified by the possession of two kinetoplasts and one nucleus (2 K 1 N) or two kinetoplasts and two nuclei (2 K 2 N) (Figures 1F to J), and were present in the midgut at both early (2–7 days) and late (12–26 days) timepoints (Table 2). Cell division was symmetrical, yielding two trypanosomes of similar size (Figure 1J).

**Table 2:** Proliferation of midgut and proventricular trypomastigotes

**Trypanosome infection**	**1 K 1 N**	**2 K 1 N**	**2 K 2 N**	**Proportion of 2 K trypanosomes**
Early midgut (days 2–7)	184	20	11	31/215 (14.4%)
Late midgut (days 12–26)	538	15	18	33/571 (5.8%)
Early proventriculus (days 6–12)	394	0	0	0/394 (0%)
Late proventriculus (days 13–26)	1266	0	0	0/1266 (0%)

Number of kinetoplasts (K) and nuclei (N) as indicated. Trypanosomes with 2 K are in the process of division.

### Proventricular trypanosomes

**Figure 2:**
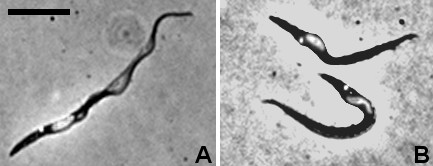
**Proventricular trypanosomes.** A. Trypomastigote with posterior kinetoplast. B. Trypomastigotes with the kinetoplast adjacent and to the posterior pole of the nucleus; the cell is distended in the region of the kinetoplast and nucleus. Bar = 10 μm.

Trypanosomes appeared in the proventriculus as early as six days after infection. Only trypomastigotes were observed (Figure 2A), although a few cells with the kinetoplast posterior but very close to the nucleus were also found; the juxtaposition of the kinetoplast and nucleus in these cells produced a marked widening or bulge (Figure 2B), reminiscent of a cell type found in the proboscis in *T. nanum* infection by Muriel Robertson [34]. The proventricular trypomastigotes appeared to be slightly longer and thinner than those in the midguts on corresponding days, and the morphology of the population remained relatively constant over time, up to the final dissection timepoint at 77 days (Additional file 3: Table S1). In contrast to the trypanosome population in the midgut, no dividing cells or 2 K 1 N/2 K 2 N cells were found in the proventricular population (Table 2).

**Figure 3:**
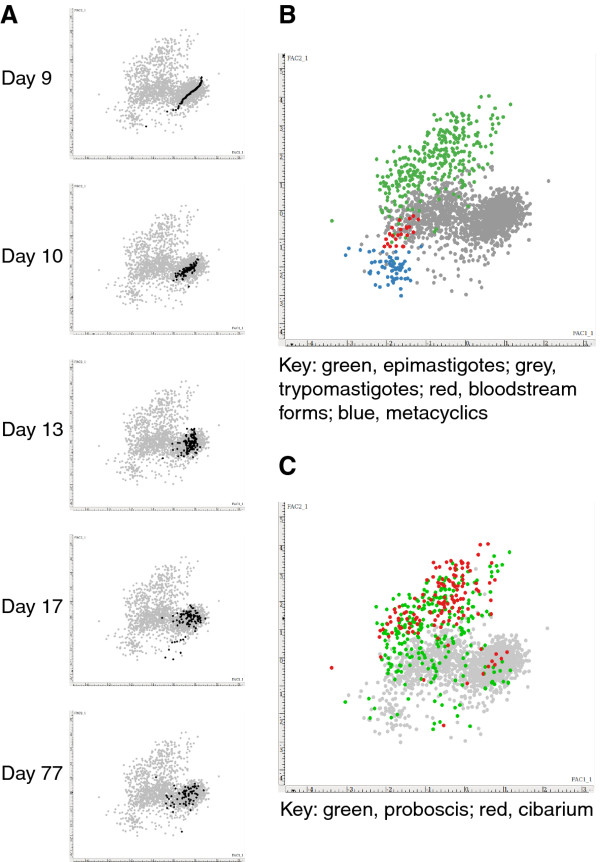
**Principal components analysis (PCA).** Each plot shows scores for PCA factor 1 versus factor 2 derived from the mensural data from 2205 individual trypanosomes, each represented by a coloured dot. A. Sequential plots of proventricular trypanosomes (black dots) compared with all other trypanosomes (grey dots). B. Comparison of bloodstream forms (red dots), midgut, proboscis and cibarium trypomastigotes (grey dots), epimastigotes (green dots), and metacyclics (blue dots). C. Comparison of trypanosomes from the proboscis and cibarium.

Principal components analysis (PCA) of the morphological parameters of the proventricular trypanosomes over time shows that they constitute a very discrete population, particularly early on in the infection time course (days 9–10, Figure 3A). Strikingly, although PCA identifies latent variables, which are uncorrelated over the whole population, there was a strong correlation between factors 1 and 2 in the proventricular trypanosomes at day 9 (Figure 3A, top panel). This reflects the fact that this population was unusually uniform in terms of the relationship between cell length and relative organelle positioning with respect to the anterior and posterior poles of the cell. The variables that contributed most to PCA factor 1 were cell length, nuclear length and the distances of the kinetoplast and nucleus from the anterior end of the cell, while for factor 2 the key variables were the distance between the kinetoplast and nucleus and the distances of the kinetoplast and nucleus from the posterior end of the cell (Additional file 2: Figure S2).

### Foregut trypanosomes

**Figure 4:**
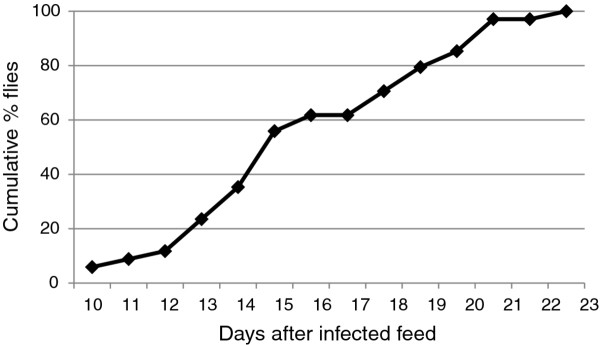
***Trypanosoma congolense*in spit samples.** Cumulative percentage of infected flies that produced a trypanosome-positive spit sample. A total of 34 flies produced a trypanosome-positive sample during the timecourse of 10–23 days.

To investigate how development proceeds beyond the proventriculus, we sampled foregut trypanosomes by inducing individually-caged flies to deposit spit samples onto glass slides from 10–23 days after infection. The spit is a mixture of saliva and regurgitated foregut contents, but only foregut trypanosomes are represented before a proboscis infection is established. Spit from two flies was already trypanosome-positive on day 10 and the number of positive flies steadily increased during the timecourse of infection (Figure 4). By day 23, 34 flies had produced at least one trypanosome-positive spit sample. On dissection, it was found that only 42 of the 50 individually-caged flies had an infected midgut and hence were capable of producing a trypanosome-positive spit sample; 39 flies had an infected proboscis (Table 1).

**Figure 5:**
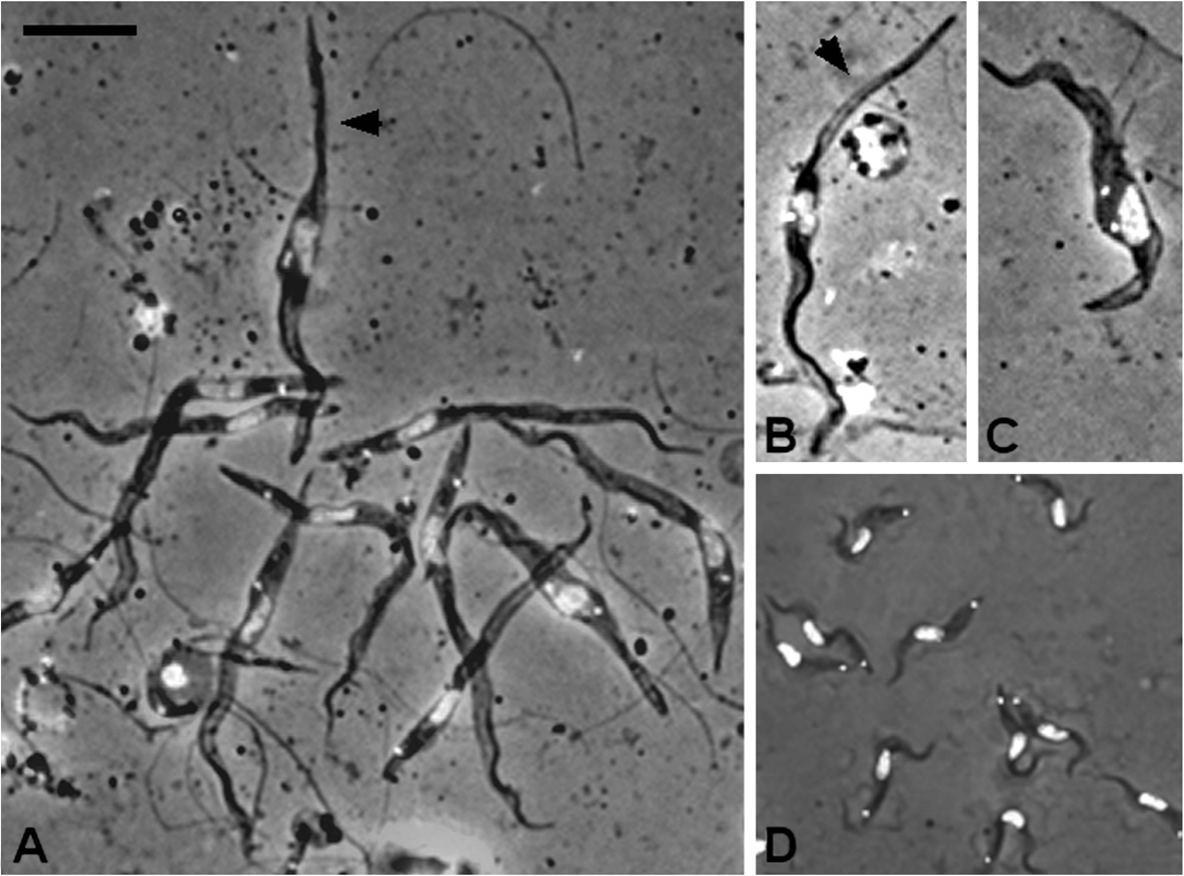
**Trypanosomes in spit samples 16–21 days after infection.** A. Mixture of trypomastigotes with epimastigote (arrowed). B. Epimastigote with long posterior (arrowed). C. Epimastigote (2K1N). D. Metacyclics from day 21; contrast the size of metacyclics with the other trypomastigotes from the proboscis shown in panel A at the same scale. Bar = 10 μm.

Initially only trypomastigotes were present in the spit samples, until epimastigotes appeared on day 15 (Figure 5A, B, C). The trypomastigotes were not noticeably different from those found in the proventriculus, except for a few very long forms found on day 16 (Additional file 4: Table S2), suggesting that the migratory trypanosomes in the foregut are simply proventricular trypanosomes that have passed through the peritrophic matrix into the foregut lumen. This contrasts with *T. brucei* where the migratory trypanosomes in the foregut also include asymmetric dividers and their daughter epimastigotes [11,16,17].

*T. congolense* proboscis infections were detected as early as day 13 by dissection, before the first appearance of epimastigotes in spit samples on day 15. This accords with previous findings that the epimastigotes arise in the proboscis, not the proventriculus or foregut [21,34]. Relatively few epimastigotes were observed in spit samples and these were highly variable in length (n = 17; Additional file 4: Table S2). Epimastigotes in the process of division (2 K 1 N) were also seen (Figure 5C). The morphology of epimastigotes in the spit and proboscis was similar. The posterior was sometimes extremely elongated, such that the nucleus was positioned equidistant between the posterior and anterior poles of the cell (Figure 5A and B); the elongated posterior sometimes twisted during fixation (Figure 5B and C). In *T. brucei*, procyclics with the so-called “nozzle phenotype”, which have a similarly elongated posterior end, have been produced *in vitro* by perturbing expression of single genes, e.g. by overexpression of the zinc finger CCCH motif protein tbZFP2 [45] or by knockdown of cyclin CYC2 [46]. Whether the nozzle phenotype is analogous in *T. brucei* procyclics and *T. congolense* epimastigotes, these experiments serve to demonstrate that relatively minor changes in gene expression are sufficient to produce the gross phenotypic changes in trypanosome length and morphology seen here.

The close proximity of the kinetoplast to the nucleus in the epimastigotes was associated with a bulge in the cell near the nucleus, particularly in dividing cells (Figures 5A-C); this was also observed for some proventricular trypomastigotes where the kinetoplast and nucleus were juxtaposed, but in that case the kinetoplast was posterior not anterior to the nucleus (Figure 3B).

Metacyclics first appeared in spit samples on day 21, identified by their very short length (12.8 ± 1.3 μm; Additional file 4: Table S2) and characteristic S-shape [31] (Figure 5D). The metacyclics were significantly shorter and thinner than BSF (Additional file 3: Tables S1, Additional file 4: Table S2); this can be clearly seen in the PCA plot in which metacyclics and BSF cluster separately (Figure 2). In metacyclics, the kinetoplast was very close to the posterior pole of the cell (Figure 5D; Additional file 4: Table S2).

### Proboscis trypanosomes

**Figure 6:**
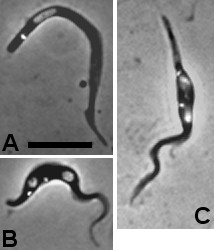
**Proboscis trypomastigotes.** A. Trypomastigote. B, C. 2K2N trypomastigotes. Bar = 10 μm.

Trypanosomes were first seen in the proboscides of flies dissected at 13 days, when long trypomastigotes and epimastigotes were present. The trypanosome population of the proboscis was highly variable in composition and morphology. The morphology of long trypomastigotes remained fairly uniform between 16 and 77 days after infection: about 30 μm in length with an elongated nucleus, and the kinetoplast and nucleus located towards the posterior of the cell (Additional file 5: Table S3); the cells were similar in morphology to those observed in spit samples (Figure 5A), suggesting that these are migratory trypanosomes from the proventriculus. Shorter trypomastigotes were also present (Figure 6); these were sometimes observed in division (Figure 6B, C), suggesting they are pre-metacyclics. Few metacyclics were recovered from proboscides at dissection and were morphologically similar to those observed in spit samples (Figure 5D; Additional file 5: Table S3).

**Figure 7:**
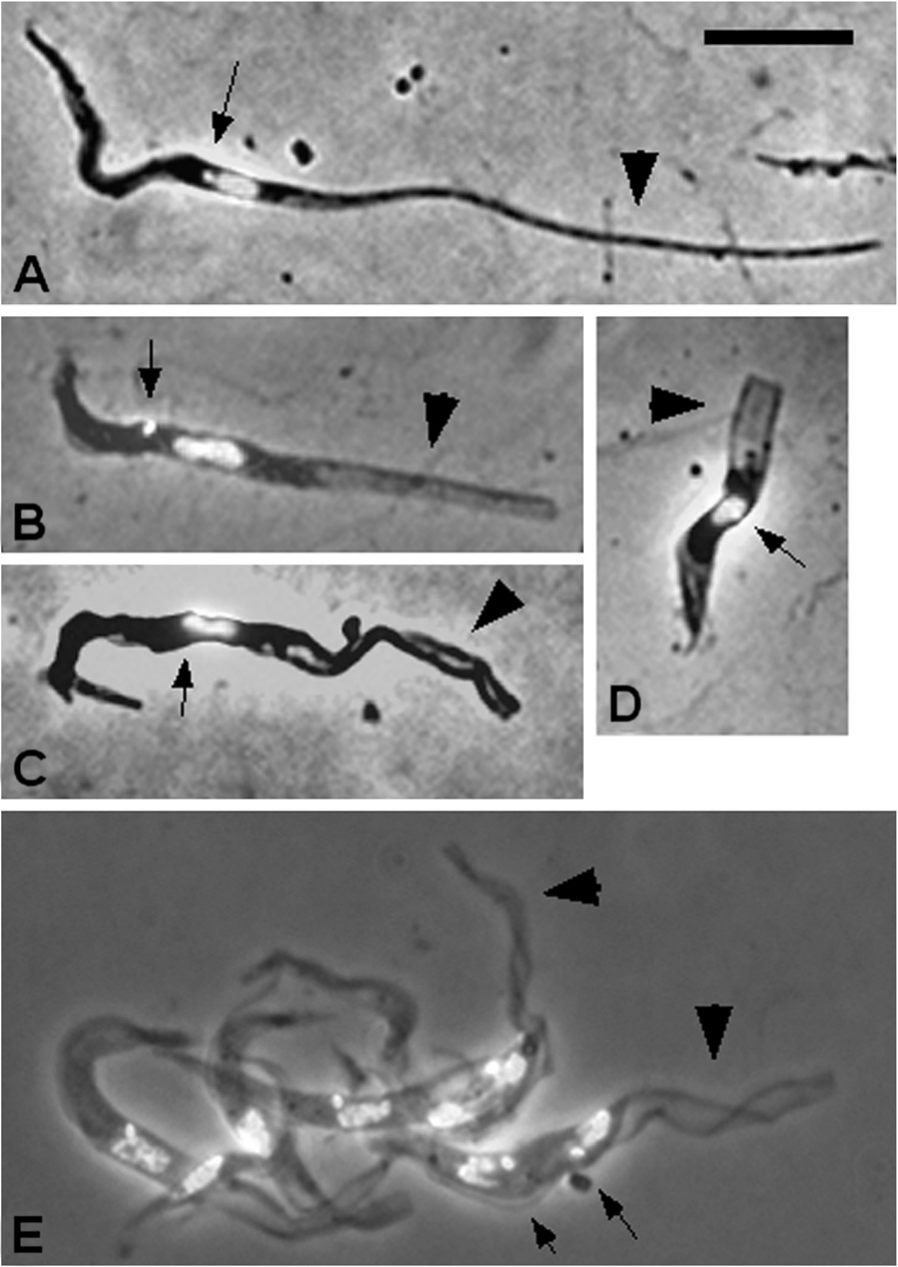
**Proboscis epimastigotes**. A. Epimastigote with long posterior (broad arrow); the kinetoplast (arrow) is adjacent and anterior to the nucleus. B - D. Epimastigotes with transparent posterior extensions (broad arrows); position of kinetoplast is indicated by thin arrow; in C the posterior extension is crumpled; D shows a truncated form. E. cluster of epimastigotes with long transparent posterior extensions; the two trypanosomes on the right have long posterior extensions (broad arrows) and are in division (2K2N); arrows indicate the kinetoplasts in the lower trypanosome. Bar = 10 μm.

The length of epimastigotes increased with duration of infection, with some extremely long cells present from day 19 onwards (Figure 7A); the average length showed a marked increase, almost doubling between days 13 and 19 (Additional file 5: Table S3). This echoes the observations from *in vitro* studies where epimastigotes are reported to contract and shorten soon after attachment to the plastic substratum and then to lengthen after a few days [26,30,31]. In live trypanosomes the elongated posterior had a rigid appearance, contrasting with the fluid undulating motion of trypomastigotes (Additional file 6: Movie 1). As in the epimastigotes from spit samples, the elongated posterior was often twisted or crumpled during fixation, and sometimes had a transparent appearance (Figure 7B, C, E). In some trypanosomes the posterior appeared to be truncated, almost looking as if the posterior was broken off or twisted back on itself (Figure 7D). Such trypanosomes were also observed *in vivo* (Additional file 7: Movie 2), so this is not an artefact of fixation.

### Cibarium trypanosomes

The cibarium is a widening of the alimentary tract that lies between the proboscis and foregut; the chitin-lined walls act as a pump allowing the fly to suck blood [47]. Both *T. congolense* and *T. vivax* are found attached to the cibarial walls [18]. Here the cibarium was examined for trypanosomes when flies were dissected at 77 days. Trypanosomes were found widely distributed across the dorsal wall as described [18] (Additional file 8: Movie 3). The majority of cibarial trypanosomes were epimastigotes (144/158, 91%), the remainder being trypomastigotes (Additional file 5: Table S3). The morphology of these cells was similar to that of trypomastigotes and epimastigotes in the proboscis, suggesting that the cibarium harbours an extension of the proboscis population rather than a separate morphological stage. This is clearly seen in the PCA comparing trypanosomes from the cibarium and proboscis; there is considerable overlap between these two groups (Figure 2).

### Transition from trypomastigote to epimastigote

The transition from trypomastigote to epimastigote involves the re-positioning of the kinetoplast relative to the nucleus. First the kinetoplast-nuclear distance diminishes before the two organelles pass by each other until the kinetoplast is fully anterior; the kinetoplast-nuclear distance then increases. As this is a gradual process, the point of transition from trypomastigote to epimastigote is uncertain. Comparison of proboscis trypanosomes at these various stages of transition shows that the longest cells are those with the kinetoplast fully anterior to the nucleus (Table 3). The increase in length is due almost entirely to growth of the posterior end of the cell, as shown by comparison of kinetoplast position relative to the posterior or anterior of the cell (Table 3). This contrasts with the transition from trypomastigote to epimastigote in *T. brucei*, which is part of an asymmetric cell division that produces one short and one long epimastigote [11]. The length of the asymmetric divider during this transition phase was fairly constant [11] and much less variable than that found here for *T. congolense* (Table 3).

**Table 3:** Measurement of proboscis epimastigotes

Measurements (μm)	Juxta-posterior	Centre	Juxta-anterior	Anterior	P-value
Cell length	23.0 ± 1.4 a	23.4 ± 1.1 a	29.0 ± 1.5 ab	30.8 ± 1.0 b	0.002
Cell width	2.2 ± 0.1 a	2.2 ± 0.1 a	2.1 ± 0.6 a	2.1 ± 0.1 a	0.709
Kinetoplast to posterior	6.8 ± 0.7 a	8.6 ± 0.6 a	14.4 ± 1.2 b	17.5 ± 0.8 b	0.001
Nucleus to posterior	6.6 ± 0.7 a	7.4 ± 0.5 a	12.0 ± 1.2 b	14.0 ± 0.8 b	0.001
Nucleus length	2.9 ± 0.2 a	2.7 ± 0.1 a	2.8 ± 0.1 a	2.9 ± 0.1 a	0.609
Nucleus width	1.2 ± 0.1 a	1.2 ± 0.1 a	1.3 ± 0.1 a	1.2 ± 0.1 a	0.393
Nucleus to anterior	16.4 ± 1.3 a	16.0 ± 0.9 a	16.9 ± 0.6 a	16.9 ± 0.4 a	0.745
Kinetoplast to anterior	16.3 ± 1.2 a	14.8 ± 0.9 a	14.6 ± 0.6 a	13.3 ± 0.4 a	0.048
N	11	28	60	79	

Mean measurements of morphological features of proboscis epimastigotes, grouped by relative position of kinetoplast to nucleus (μm ± s.e.m.). Statistics were done for each feature, by row, for differences between relative positions of kinetoplast and nucleus (ANOVA, P-value for the overall model in last column). Means which share the same letter (a or b) are not significantly different by post-hoc testing (P ≤ 0.05). N = number of trypanosomes.

**Figure 8:**
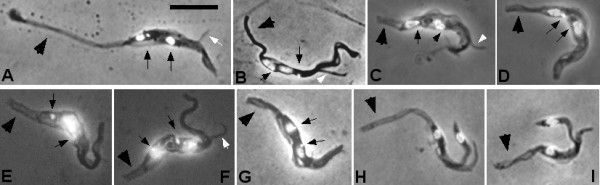
**Proboscis dividing trypanosome.** Fixed and DAPI-stained trypanosomes in division (2K1N or 2K2N) from dissected proboscides; these trypanosomes were free rather than attached inside the proboscis. Panels A-C show examples of epimastigotes apparently giving rise to daughter epimastigotes. Panels D, F and G show examples of trypomastigotes apparently giving rise to daughter epimastigotes. Panel E shows an epimastigote apparently giving rise to a daughter trypomastigote. Panels H and I show epimastigotes apparently giving rise to daughter trypomastigotes in an asymmetric division. Positions of kinetoplasts are indicated by thin arrows; posterior indicated by broad arrow; daughter flagellum indicated by white arrow. Bar = 10 μm.

We searched among fixed and DAPI-stained trypanosomes from dissected proboscides for 2 K 1 N/2 K 2 N and dividing trypanosomes (Figure 8A-I); note that this sample represents trypanosomes that were free rather than attached inside the proboscis. Figures 8A-C show examples of epimastigotes apparently giving rise to daughter epimastigotes; in some cases the daughter epimastigote clearly had a very long posterior (Figures 8A), suggesting that division may be asymmetric, though the length of the posterior of the parental cell is hard to judge. Figures 8D, F and G show examples of trypomastigotes apparently giving rise to daughter epimastigotes with posterior ends of modest length; we assume these dividing stages show the transition from proventricular trypomastigotes to epimastigotes that will subsequently attach to the lining of the proboscis, but are cautious of constructing a narrative from a few fixed cells. In contrast, Figure 8E shows an example of the reverse, an epimastigote giving rise to a daughter trypomastigote; this can be interpreted as the first step on the pathway to metacyclic, again with the caveat that these were rarely observed, fixed cells. The same caveat applies to the curious asymmetric pairs of cells, which also appear to show epimastigotes giving rise to daughter trypomastigotes, but in these examples, very long epimastigotes and very short trypomastigotes (Figures 8H and 8I). The scarcity of dividing stages, coupled with the fact that we sampled only unattached trypanosomes spilt from the proboscis, means that we are unable to state categorically that the transition from trypomastigote to epimastigote, and subsequently from epimastigote to trypomastigote, is always associated with cell division.

## Discussion

**Figure 9:**
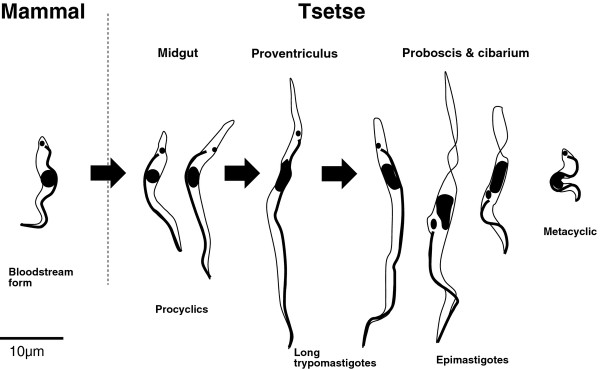
***T. congolense*life cycle stages.** Representative life cycle stages are shown in their respective locations in the mammalian or tsetse hosts. Bloodstream forms taken up by the fly (arrow) differentiate to procyclics in the fly midgut and grow in length. In the proventriculus the procyclics cease division and become uniform in size and shape. These trypomastigotes migrate to the cibarium and proboscis, where they differentiate to epimastigotes; some of these forms have extremely long or truncated posterior ends as shown in these examples. The infective metacyclics are very small and do not divide. The exact sequence of events between proventricular trypomastigotes arriving in the proboscis/cibarium and production of metacyclics is uncertain, and whether there are gradual or abrupt transitions between stages remains to be elucidated.

We present a detailed picture of the developmental cycle of *T. congolense* in the tsetse fly vector, which is summarised in Figure 9. Besides the basic fact that infective metacyclics of *T. congolense* are found in the proboscis while those of *T. brucei* are in the salivary glands, there are also significant differences in the forms that migrate anteriorly from the midgut to the mouthparts. This is surprising considering the intrinsic biological similarity of the two species and their close phylogenetic relationship. Moreover, the initial phase of development as midgut procyclics is much the same in the two species. Bloodstream forms shed their variant surface glycoprotein (VSG) surface coat and differentiate into elongated proliferative forms in the remains of the bloodmeal. These procyclics pass through the peritrophic matrix (PM) into the ectoperitrophic space before migrating anteriorly to colonize the proventriculus, where they cease division. In *T. congolense* the proventricular trypomastigotes constitute a morphologically discrete population; we observed that PCA factors 1 and 2 were highly correlated in proventricular trypomastigotes, while they were uncorrelated in the total population (by definition), indicating that the proventricular trypomastigotes are subject to morphological constraints in *T. congolense*. These long proventricular trypomastigotes pass through the PM [35] to enter the foregut lumen before migrating to the proboscis. Here they become epimastigotes and attach to the chitinous lining of the proboscis and cibarium [18], where they proliferate and subsequently develop into infective metacyclics [22]. In contrast, for *T. brucei* the proventricular stage is arrested in G2 with a 4 N DNA content, and subsequently divides asymmetrically into one short and one long epimastigote; all these forms can be found in the foregut contents of the fly as they migrate anteriorly [11,16,17].

Thus the process of differentiating from trypomastigote to epimastigote is radically different in the two species. In *T. brucei* this is achieved by an asymmetric division of the proventricular trypomastigote; the kinetoplast migrates round the nucleus towards the anterior before dividing, so that two epimastigote daughter cells are formed when the nucleus divides [11]. We found no evidence of such a well orchestrated transition in *T. congolense.* A range of long trypanosomes with the kinetoplast positioned adjacent to the nucleus were recovered from the proboscis; by subdividing these according to the relative positions of the kinetoplast and nucleus, we infer that the transition from trypomastigote to epimastigote involves both migration of the kinetoplast around the nucleus to an anterior position and elongation of the posterior of the trypanosome. In the longest epimastigotes, the anterior kinetoplast was well separated from the nucleus. The re-positioning of the kinetoplast relative to the nucleus and remodelling of the cell posterior may occur without cell division, but we also found examples of dividing forms in transition from epimastigotes to trypomastigotes and *vice versa*.

Seen by scanning electron microscopy (SEM), attached epimastigotes of *T. brucei* are of fairly uniform size and possess an elongated thin posterior that protrudes into the lumen of the salivary gland [43]. In contrast, *T. congolense* epimastigotes are highly variable in morphology. Some are spectacularly long with elongated twisted or transparent posterior extensions, much broader than the thin, pointed “nozzles” of *T. brucei* epimastigotes. Others appear truncated, the posterior of the cell with a blunt rather than tapering end, almost as if the end has been broken off or folded back on itself. Such truncated forms have been described by several workers [20,21,31]; both truncated and elongated forms are visible in SEM images of *in vitro* cultivated epimastigotes [31]. Here truncated forms were observed as live, motile cells and they are clearly not an artefact of fixation.

The extreme length of *T. congolense* epimastigotes prompts questions of how they differentiate into metacyclics, which involves a gross reduction in cell length from about 30 μm to 13 μm. In cultures of long attached epimastigotes, metacyclics only appeared if short, attached trypomastigotes were present, suggesting that these are a transitional stage [31]. *In vivo*, VSG-coated trypanosomes were found in both the hypopharynx and occasionally the labrum by transmission EM [22], supporting the view that differentiation to metacyclics occurs predominantly in the hypopharynx [2,21]. Here, the hypopharynx trypanosomes were distinguishable in live dissected material, but were mixed with those from the labrum during fixation and staining; attached cells were probably under-represented. In such preparations both long and truncated epimastigotes were observed in division, as well as trypomastigotes. As far as could be discerned, these divisions rarely resulted in large reduction in length of the daughter cell, although in the early stage of division before the cleavage furrow develops, it is not possible to judge the length of the posterior of the daughter cell. The short dividing trypomastigotes of about 20 μm in length were assumed to be pre-metacyclics and fit the description of the transitional stage observed *in vitro*[31]. As far as can be ascertained, *in vitro* production of *T. congolense* metacyclics follows the same developmental pathway as in the fly [26,28,30,31]; however, there may be subtle differences in terms of gene expression, for example of surface molecules, that will only be evident from detailed investigation.

Both *T. congolense* and *T. brucei* share the same migration route in the tsetse fly from the midgut to the mouthparts via the proventriculus and foregut, but the two strains used here evidently differ in their ability to complete the developmental cycle in *G. m. morsitans*. Established midgut infections led to far more proboscis infections in *T. congolense* than salivary gland infections in *T. brucei* (transmission indices of 93% and 25% respectively). Invasion of the foregut was equally efficient in both species, as judged by the percentage of spit-positive/midgut-positive flies (81% v. 83%). While most of these foregut infections led to the successful invasion and colonisation of the proboscis by *T. congolense*, relatively few salivary gland infections were established by *T. brucei* (100% v. 30%). Surface coat proteins and carbohydrates are thought to play a protective role against insect innate immune responses [48], so although these molecules differ between the two species, they evidently offer equivalent levels of protection while the parasites are in the midgut, proventriculus and foregut. The problem comes when the parasites reach the mouthparts: for *T. congolense* it is clearly quite easy to settle down and proliferate in the proboscis, but the migratory forms of *T. brucei* pass through this region to enter the salivary glands via the hypopharynx. The attrition rate is high and it seems that only a few trypanosomes initiate the infection [41,49]. The evolutionary driver of this strategy in *T. brucei* was presumably competition for space and nutrients, because several different trypanosome species attach in the proboscis (*T. congolenseT. vivaxT. simiae* and *T. godfreyi*). By-passing this region opens the greater surface area of the salivary glands for colonisation.

The trypanosome strain used here represents the savannah subgroup of *T. congolense*. Within subgenus *Nannomonas*, there are an additional two subgroups of *T. congolense* (forest and Kenya Coast or kilifi), plus *T. simiae* and *T. godfreyi* and related trypanosomes [50]. Comparative analysis will show how far this description of the life cycle of *T. congolense* savannah generalizes to the whole subgenus.

## Conclusions

We have presented a detailed description of the life cycle of *T. congolense* in its tsetse fly vector. This comprehensive account has allowed comparison with the better known life cycle of *T. brucei*. These related trypanosomes share a common migration pathway during development in the fly, involving the establishment of infection in the ectoperitrophic space of the midgut and invasion of the proventriculus. After this, the transitional developmental stages in the foregut and mouthparts are remarkably different, before the life cycles converge again to culminate in the production of infective metacyclics.

## Competing interests

The authors declare that they have no competing interests.

## Authors' contributions

WG, LP and MB designed the study. SC, LP and VF carried out the tsetse transmission experiments and imaging; LP and MB carried out the statistical analyses; WG, LP and MB drafted the manuscript. All authors read and approved the final manuscript.

## Supplementary Material

Additional file 1 **Figure S1.** Diagram of measurements. Diagram of measurements made on *Trypanosoma congolense* cells found in tsetse flies. The distance from the kinetoplast to the anterior (Kant) was calculated from L - Kpost. Similarly, the distance from the nucleus to the anterior (Nant) = L - NPost. The distance from the kinetoplast to the posterior edge of the nucleus (KNuc) is positive when the kinetoplast is posterior to the nucleus and negative when it is anterior to the nucleus. Click here for file

Additional file 2 **Figure S2.** Loadings for PCA factors 1 and 2. Plot of absolute values of loadings for PCA factors 1 and 2. Measurements as defined in Additional file 6: Figure S1; values were log transformed unless normally distributed. The variables that contribute most to PCA factor 1are logL, logKAnt, logNAnt and logNL, i.e. factor 1 reflects primarily cell length, nuclear length and the distances of the kinetoplast and nucleus from the anterior end of the cell. The variables that contribute most to PCA factor 2 are logKPost, logNPost and KNuc, i.e. factor 2 represents primarily the distance between the kinetoplast and nucleus and the distances of the kinetoplast and nucleus from the posterior end of the cell. Click here for file

Additional file 3 **Table S1.** Morphometry of *T. congolense* cells found in blood or tsetse midgut and proventriculus. The mean ± SEM in μm is top line in each box with the range below. Click here for file

Additional file 4 **Table S2.** Morphometry of *T. congolense* cells found in spit samples. The mean ± SEM in μm is top line in each box with the range below. Click here for file

Additional file 5 **Table S3.** Morphometry of *T. congolense* cells found in the proboscis and cibarium. The mean ± SEM in μm is top line in each box with the range below. Click here for file

Additional file 6 **Movie 1.** Long epimastigote from proboscis (Long_epi.mov). Trypanosomes from a proboscis dissected 13 days after infection. Upper left, sinuous trypomastigote; lower centre, long epimastigote with rigid posterior extension. Click here for file

Additional file 7 **Movie 2.** Epimastigotes from proboscis (v2-1.mov). Cluster of 6 long epimastigotes; the cell on the right has a truncated posterior extension. Click here for file

Additional file 8 **Movie 3.** Cibarium trypanosomes (Cibarium.mov). Trypanosomes in the cibarium. Click here for file
