# Supplementary figures and images for: The life cycle of Trypanosoma (Nannomonas) congolense in the tsetse fly
Source: Parasit Vectors. 2012 Jun 27;5:109. doi: 10.1186/1756-3305-5-109 (PMC3384477; doi:10.1186/1756-3305-5-109)

## Slide 1
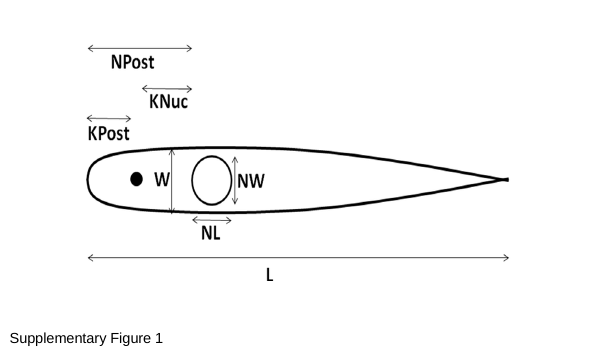

Supplementary Figure 1

Supplement: Additional file 1 — Figure S1. Diagram of measurements. Diagram of measurements made on Trypanosoma congolense cells found in tsetse flies. The distance from the kinetoplast to the anterior (Kant) was calculated from L - Kpost. Similarly, the distance from the nucleus to the anterior (Nant) = L - NPost. The distance from the kinetoplast to the posterior edge of the nucleus (KNuc) is positive when the kinetoplast is posterior to the nucleus and negative when it is anterior to the nucleus. [file 1756-3305-5-109-S1.ppt]
