# Supplementary material for: The life cycle of Trypanosoma (Nannomonas) congolense in the tsetse fly
Source: Parasit Vectors. 2012 Jun 27;5:109. doi: 10.1186/1756-3305-5-109 (PMC3384477; doi:10.1186/1756-3305-5-109)

	Factor 1	Factor 2
**Knuc**	0.162	-0.485
**logL**	0.457	0.195
**logW**	-0.238	0.311
**logKPost**	0.145	0.578
**logNPost**	0.236	0.471
**logNL**	0.416	-0.056
**logNW**	-0.210	0.205
**logKAnt**	0.449	-0.167
**logNAnt**	0.461	0.023

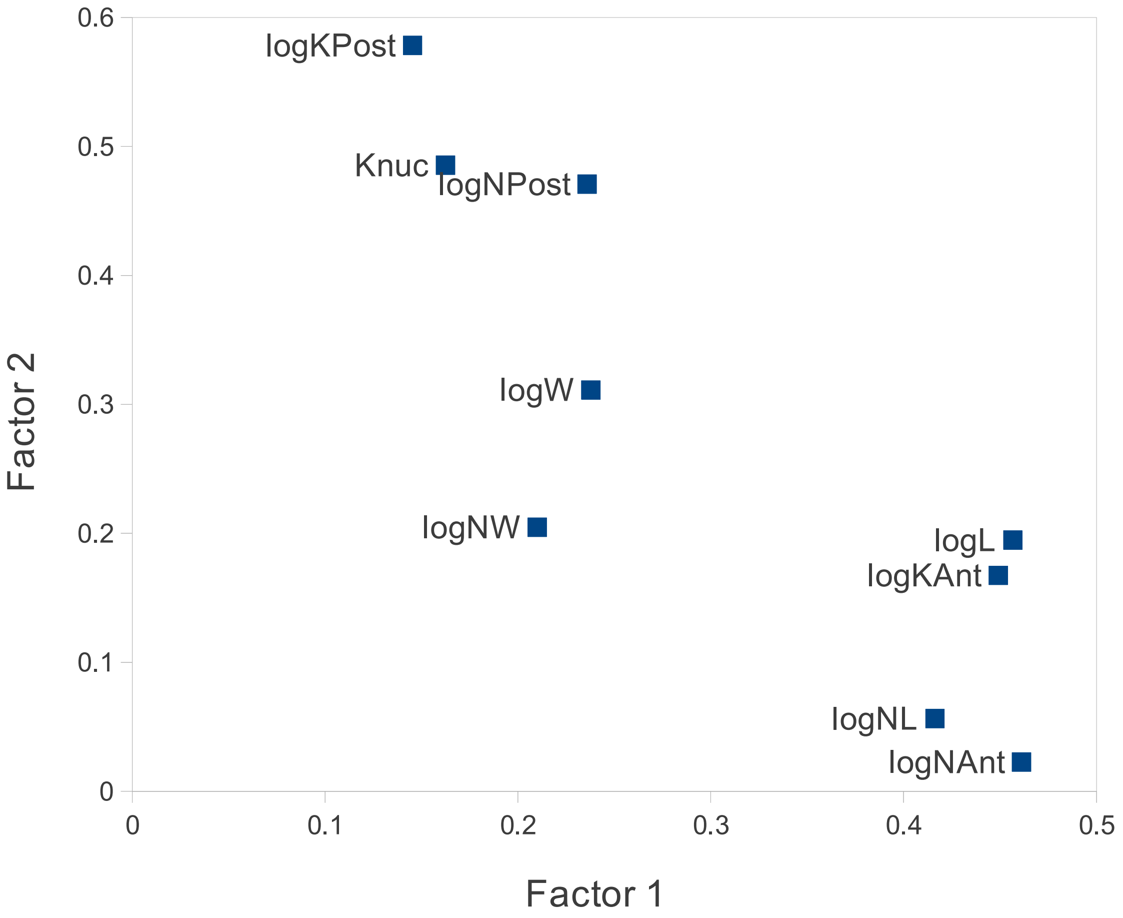

Supplement: Additional file 2 — Figure S2. Loadings for PCA factors 1 and 2. Plot of absolute values of loadings for PCA factors 1 and 2. Measurements as defined in Additional file 6: Figure S1; values were log transformed unless normally distributed. The variables that contribute most to PCA factor 1are logL, logKAnt, logNAnt and logNL, i.e. factor 1 reflects primarily cell length, nuclear length and the distances of the kinetoplast and nucleus from the anterior end of the cell. The variables that contribute most to PCA factor 2 are logKPost, logNPost and KNuc, i.e. factor 2 represents primarily the distance between the kinetoplast and nucleus and the distances of the kinetoplast and nucleus from the posterior end of the cell. [file 1756-3305-5-109-S2.pdf]
