# Supplementary material for: The life cycle of Trypanosoma (Nannomonas) congolense in the tsetse fly
Source: Parasit Vectors. 2012 Jun 27;5:109. doi: 10.1186/1756-3305-5-109 (PMC3384477; doi:10.1186/1756-3305-5-109)
Supplement: Additional file 3 — Table S1. Morphometry of T. congolense cells found in blood or tsetse midgut and proventriculus. The mean ± SEM in μm is top line in each box with the range below. [file 1756-3305-5-109-S3.doc]

**Table S1. Morphometry of *T. congolense* cells in blood, tsetse midgut and proventriculus**. The mean ± SE in µm is top line in each box with the range below.

| Morphotype | Timepoint (Number) | L | W | KPost | KNuc | NPost | NL | NW | KAnt | NAnt |
| --- | --- | --- | --- | --- | --- | --- | --- | --- | --- | --- |
| Bloodstream forms | Day 3  (23) | 17.47 ± 0.39 14.37-21.38 | 2.55 ± 0.07 2.05-3.28 | 1.08 ± 0.07 0.49-1.64 | 3.44 ± 0.13 2.15-4.72 | 5.28 ± 0.16 3.92-6.35 | 2.47 ± 0.08 1.93-3.17 | 1.60 ± 0.06 1.16-2.11 | 16.39 ± 0.35 13.47-19.74 | 12.19 ± 0.30 9.89-15.18 |
| Midgut trypomastigotes | Day 2  (77) | 20.00 ± 0.35 13.16-26.24 | 2.62 ± 0.06 1.67-3.87 | 3.00 ± 0.15 0.08-6.43 | 2.07 ± 0.10 0.41-3.91 | 5.95 ± 0.19 2.44-9.39 | 2.92 ± 0.07 1.60-4.59 | 1.73 ± 0.04 0.87-2.47 | 17.01 ± 0.27 12.24-22.16 | 14.06 ± 0.25 9.94-19.97 |
|  | Day 3.  (42) | 20.68 ± 0.52 13.01-26.90 | 2.20 ± 0.06 1.32-2.93 | 3.06 ± 0.20 1.13-7.17 | 2.29 ± 0.11 0.92-3.94 | 6.19 ± 0.23 2.58-10.49 | 2.72 ± 0.06 2.12-3.45 | 1.42 ± 0.04 0.90-2.11 | 17.74 ± 0.42 11.88-23.46 | 14.62 ± 0.37 10.29-19.49 |
|  | Day 4  (75) | 21.90 ± 0.39 14.34-30.08 | 1.94 ± 0.04 1.04-2.76 | 3.06 ± 0.14 1.06-6.30 | 2.61 ± 0.09 0.77-4.62 | 6.46 ± 0.19 2.74-11.64 | 3.01 ± 0.06 1.67-4.91 | 1.54 ± 0.30 0.65-2.19 | 18.85 ± 0.30 12.90-25.53 | 15.45 ± 0.28 9.83-20.93 |
|  | Day 5 (102) | 23.38 ± 0.38 13.80-36.07 | 1.94 ± 0.03 1.32-2.87 | 4.22 ± 0.18 0.33-7.67 | 2.29 ± 0.08 0.65-4.31 | 7.43 ± 0.18 2.98-11.17 | 3.13 ± 0.05 2.09-5.05 | 1.43 ± 0.02 0.59-2.11 | 19.16 ± 0.31 12.54-32.60 | 15.94 ± 0.30 10.02-29.52 |
|  | Day 6 (111) | 23.30 ± 0.33 12.76-30.54 | 1.88 ± 0.03 1.15-3.39 | 3.47 ± 0.14 0.16-8.43 | 2.67 ± 0.06 1.32-4.66 | 7.10 ± 0.15 3.70-12.05 | 2.98 ± 0.05 1.92-4.16 | 1.43 ± 0.02 1.06-1.92 | 19.84 ± 0.27 12.03-27.38 | 16.20 ± 0.25 8.68-23.62 |
|  | Day 9 (141) | 33.58 ± 0.46 20.86-45.25 | 1.74 ± 0.03 0.92-2.93 | 4.34 ± 0.16 0.88-16.18 | 3.85 ± 0.08 1.38-6.97 | 9.16 ± 0.17 4.03-20.96 | 4.08 ± 0.06 2.19-5.78 | 1.17 ± 0.03 0.59-2.15 | 29.24 ± 0.39 16.76-38.60 | 24.43 ± 0.39 13.49-34.68 |
|  | Day 10 (111) | 32.71 ± 0.78 15.05-49.25 | 1.66 ± 0.04 0.91-2.84 | 4.76 ± 0.21 0.49-9.87 | 2.75 ± 0.09 0.91-6.21 | 8.49 ± 0.22 3.27-12.86 | 3.86 ± 0.07 2.16-5.67 | 1.25 ± 0.03 0.63-2.03 | 27.96 ± 0.65 12.90-39.84 | 24.22 ± 0.65 11.19-36.76 |
|  | Day 13 (110) | 34.35 ± 0.55 21.13-45.05 | 1.65 ± 0.04 0.93-2.78 | 4.94 ± 0.16 0.54-9.08 | 2.80 ± 0.08 0.88-6.65 | 8.69 ± 0.16 4.36-14.01 | 4.38 ± 0.07 2.80-6.54 | 1.12 ± 0.02 0.65-1.80 | 29.41 ± 0.55 16.21-39.17 | 25.66 ± 0.52 13.76-35.63 |
|  | Day 17 (192) | 40.89 ± 0.36 27.69-51.91 | 1.31 ± 0.02 0.86-2.98 | 5.22 ± 0.11 0.34-11.13 | 2.66 ± 0.04 1.48-5.01 | 8.78 ± 0.11 3.75-13.97 | 4.30 ± 0.03 2.94-5.52 | 0.99 ± 0.01 0.57-1.54 | 35.67 ± 0.31 25.15-45.55 | 32.11 ± 0.32 20.99-42.13 |
| Proventricular trypomastigotes | Day 9 (168) | 38.04 ± 0.39 15.92-48.34 | 1.51 ± 0.02 0.93-2.61 | 5.00 ± 0.13 0.18-9.78 | 3.61 ± 0.06 1.31-5.69 | 9.62 ± 0.13 4.73-13.73 | 4.34 ± 0.05 2.60-6.34 | 1.08 ± 0.02 0.63-1.93 | 33.03 ± 0.27 15.74-39.12 | 28.42 ± 0.27 11.19-34.61 |
|  | Day 10 (93) | 35.69 ± 0.44 25.05-44.38 | 1.47 ± 0.03 0.87-2.60 | 4.23 ± 0.14 0.23-7.03 | 2.63 ± 0.08 0.92-4.44 | 7.75 ± 0.15 4.48-11.79 | 4.32 ± 0.05 3.32-5.83 | 1.04 ± 0.02 0.63-1.38 | 31.46 ± 0.48 20.52-40.26 | 27.94 ± 0.30 20.57-32.59 |
|  | Day 13 (123) | 37.18 ± 0.33 25.30-45.85 | 1.59 ± 0.04 0.93-3.27 | 4.94 ± 0.12 1.67-8.42 | 2.35 ± 0.07 0.24-4.52 | 8.16 ± 0.12 3.94-11.78 | 4.46 ± 0.06 2.30-5.65 | 0.99 ± 0.02 0.57-1.67 | 32.23 ± 0.27 21.22-39.61 | 29.02 ± 0.27 19.99-36.54 |
|  | Day 17 (101) | 37.70 ± 0.50 26.77-47.30 | 1.62 ± 0.03 0.89-2.88 | 4.50 ± 0.18 0.08-9.37 | 2.57 ± 0.08 1.03-4.66 | 7.85 ±0.20 2.18-12.16 | 4.28 ± 0.05 3.05-5.53 | 1.10 ± 0.02 0.73-1.62 | 33.20 ± 0.45 23.31-42.74 | 29.86 ± 0.44 18.81-39.62 |
|  | Day 77 (69) | 36.21 ± 0.57 24.21-47.16 | 1.84 ± 0.04 0.91-2.70 | 4.31 ± 0.22 0.16-7.84 | 2.59 ± 0.09 0.41-4.24 | 7.52 ± 0.24 3.50-11.57 | 3.92 ± 0.06 2.70-4.86 | 0.98 ± 0.03 0.51-1.40 | 31.90 ± 0.46 16.55-40.20 | 28.69 ± 0.44 15.15-37.52 |
