# Supplementary material for: The life cycle of Trypanosoma (Nannomonas) congolense in the tsetse fly
Source: Parasit Vectors. 2012 Jun 27;5:109. doi: 10.1186/1756-3305-5-109 (PMC3384477; doi:10.1186/1756-3305-5-109)
Supplement: Additional file 4 — Table S2. Morphometry of T. congolense cells found in spit samples. The mean ± SEM in μm is top line in each box with the range below. [file 1756-3305-5-109-S4.doc]

Table S2. Morphometry of *T. congolense* cells found in spit samples. The mean ± SE in µm is top line in each box with the range below.

| Morphotype | Timepoint (Number) | L | W | KPost | KNuc | NPost | NL | NW | KAnt | NAnt |
| --- | --- | --- | --- | --- | --- | --- | --- | --- | --- | --- |
| Spit trypomastigotes | Day 13  (3) | 29.87 ± 3.97 25.09-37.75 | 2.58 ± 0.10 2.37-2.71 | 1.92 ± 1.05 0.08-3.72 | 2.81 ± 0.42 2.24-3.62 | 5.58 ± 0.09 4.25-7.37 | 4.09 ± 0.26 3.57-4.38 | 1.64 ± 0.10 1.45-1.79 | 27.95 ± 3.04 24.82-34.03 | 24.29 ± 3.06 20.84-30.38 |
|  | Day 14 (49) | 31.48 ± 0.57 20.37-40.62 | 2.13 ± 0.06 1.35-3.13 | 2.23 ± 0.16 0.49-5.68 | 3.59 ± 0.11 1.78-4.90 | 6.31 ± 0.22 3.47-10.07 | 3.56 ± 0.09 2.11-4.64 | 1.39 ± 0.05 0.73-2.76 | 29.26 ± 0.55 18.33-39.32 | 25.17 ± 0.53 14.24-36.00 |
|  | Day 15 (16) | 29.76 ± 1.56 14.58-38.20 | 1.86 ± 0.08 1.34-2.26 | 2.12 ± 0.36 0.33-4.61 | 3.52 ± 0.36 0.59-6.19 | 6.22 ± 0.50 2.11-11.00 | 3.57 ± 0.15 2.84-4.92 | 1.61 ± 0.06 1.28-2.01 | 27.64 ± 1.54 12.27-35.34 | 23.54 ± 1.28 11.01-31.09 |
|  | Day 16 (52) | 38.44 ± 0.91 25.11-69.21 | 2.10 ± 0.07 1.09-3.42 | 4.94 ± 0.40 0.49-15.58 | 3.19 ± 0.16 0.32-6.12 | 8.72 ± 0.33 5.53-19.86 | 4.13 ± 0.09 1.96-5.45 | 1.49 ± 0.05 0.77-2.38 | 33.50 ± 0.79 20.57-53.63 | 29.71 ± 0.74 18.50-49.35 |
| Total | Days 13-16 (120) | 34.22 ±0.61 14.58-69.21 | 2.10 ± 0.04 1.09-3.42 | 3.38 ± 0.23 0.08-15.58 | 3.39 ±0.10 0.32-6.19 | 7.32 ± 0.21 2.10-19.86 | 3.82 ± 0.06 1.96-5.45 | 1.46 ± 0.03 0.73-2.76 | 30.85 ±0.51 12.27-53.63 | 26.90 ± 0.48 11.01-49.35 |
| Spit epimastigotes | Day 15 (10) | 20.30 ± 1.14 15.94-26.56 | 1.74 ± 0.18 0.97-2.52 | 4.53 ± 0.92 1.33-7.44 | -2.72 ± 0.36 -3.78--1.27 | 2.60 ± 0.36 0.54-3.88 | 3.11 ± 0.28 1.95-5.21 | 1.98 ± 0.14 0.85-2.18 | 13.64 ± 0.99 10.79-17.96 | 17.70 ± 0.96 14.57-23.51 |
|  | Day 16  (7) | 34.78 ± 3.24 21.69-44.01 | 2.75 ± 0.22 1.84-3.73 | 14.08 ± 2.62 6.23-22.93 | -1.86 ± 0.38—3.27—0.751 | 11.27 ± 2.33 3.59-19.66 | 4.41 ± 0.30 2.81-5.36 | 1.59 ± 0.12 1.24-2.04 | 22.89 ± 1.49 16.55-26.39 | 23.51 ± 1.70 16.13-27.14 |
| Total | Days 15-16 (17) | 26.26 ±2.29 15.94-44.01 | 2.16 ± 0.18 0.97-3.73 | 9.30 ± 1.95 1.31-22.93 | -2.29 ± 0.28 -3.78--0.75 | 6.17 ± 1.42 0.54-19.66 | 3.64 ± 0.26 1.95-5.36 | 1.47 ± 0.10 0.85-2.18 | 18.26 ± 1.63 10.79-26.39 | 20.09 ± 1.12 14.57-27.14 |
| Spit  metacyclics | Days 21 & 41 (60) | 12.81 ± 1.27 10.58-18.79 | 1.28 ± 0.37 0.73-2.73 | 0.56 ± 0.30 0.16-1.82 | 3.69 ± 0.83 1.42-6.22 | 4.84 ± 0.84 2.86-7.98 | 2.41 ± 0.34 1.50-2.94 | 0.95 ± 0.20 0.52-1.63 | 12.25 ± 1.17 9.89-16.97 | 7.97 ± 1.27 5.47-14.47 |
