# Supplementary material for: The life cycle of Trypanosoma (Nannomonas) congolense in the tsetse fly
Source: Parasit Vectors. 2012 Jun 27;5:109. doi: 10.1186/1756-3305-5-109 (PMC3384477; doi:10.1186/1756-3305-5-109)
Supplement: Additional file 5 — Table S3. Morphometry of T. congolense cells found in the proboscis and cibarium. The mean ± SEM in μm is top line in each box with the range below. [file 1756-3305-5-109-S5.doc]

Table S3. Morphometry of *T. congolense* cells found in the proboscis and cibarium. The mean ± SE in µm is top line in each box with the range below.

| Morphotype | Timepoint (Number) | L | W | KPost | KNuc | NPost | NL | NW | KAnt | NAnt |
| --- | --- | --- | --- | --- | --- | --- | --- | --- | --- | --- |
| Proboscis trypomastigotes | Day 13 (14) | 22.17 ± 1.34 16.15-34.61 | 2.30 ± 0.16 1.08-3.52 | 2.20 ± 0.33 0.08-4.08 | 2.12 ± 0.16 1.40-3.36 | 4.88 ± 0.39 2.55-7.45 | 2.53 ± 0.19 1.46-3.70 | 1.17 ± 0.10 0.69-1.92 | 19.97 ± 1.42 13.62-30.90 | 17.29 ± 1.36 11.44-27.16 |
|  | Day 16 (32) | 28.39 ± 1.39 17.14-42.07 | 1.94 ± 0.10 0.92-3.05 | 3.70 ± 0.27 0.41-6.87 | 1.85 ± 0.16 0.32-3.47 | 6.33 ± 0.34 1.91-9.12 | 3.05 ± 0.13 1.75-4.43 | 1.26 ± 0.08 0.54-2.09 | 24.69 ± 1.43 14.57-37.21 | 22.06 ± 1.33 12.50-34.40 |
|  | Day 19 (7) | 29.77 ± 2.18 22.80-40.67 | 2.06 ± 0.11 1.61-2.40 | 2.44 ± 0.98 0.08-6.38 | 2.75 ± 0.27 2.04-3.99 | 6.04 ± 1.08 3.21-9.94 | 3.37 ± 0.18 2.73-4.12 | 1.18 ± 0.14 0.65-1.75 | 27.33 ± 1.85 21.71-35.72 | 23.73 ± 1.80 17.55-31.12 |
|  | Day 24 (2) | 24.02 ± 0.36 23.66-24.37 | 2.58 ± 0.15 2.44-2.73 | 2.90 ± 0.26 2.64-3.16 | 1.60 ± 0.62 0.98-2.21 | 4.79 ± 0.48 4.31-5.27 | 3.32 ± 0.20 3.12-3.52 | 1.19 ± 0.24 0.95-1.42 | 21.12 ± 0.10 21.02-21.21 | 19.23 ± 0.13 19.10-19.35 |
|  | Day 28 (6) | 25.22 ± 2.68 18.90-37.47 | 2.04 ± 0.10 1.72-2.31 | 4.00 ± 0.54 1.93-5.37 | 1.29 ± 0.33 0.41-2.38 | 5.88 ± 0.32 4.75-6.69 | 3.39 ± 0.43 1.95-5.12 | 1.07 ± 0.08 0.77-1.31 | 21.22 ± 3.00 15.43-35.54 | 19.34 ± 2.86 12.64-32.72 |
|  | Day 31 (5) | 30.42 ± 3.91 19.00-43.03 | 2.02 ± 0.22 1.32-2.66 | 5.81 ± 2.20 1.90-13.51 | 1.25 ± 0.46 0.08-2.48 | 7.53 ± 1.88 4.06-14.56 | 3.48 ± 0.36 2.16-4.23 | 1.13 ± 0.03 1.06-1.22 | 24.61 ± 3.44 11.19-29.72 | 22.89 ± 3.06 11.16-28.47 |
|  | Day 77 (13) | 30.72 ± 1.86 17.30-38.73 | 2.50 ± 0.19 1.77-3.31 | 4.71 ± 0.88 0.08-10.24 | 1.35 ± 0.28 0.18-3.54 | 6.56 ± 0.77 2.45-11.84 | 3.85 ± 0.23 2.52-5.21 | 1.27 ± 0.08 0.93-1.92 | 26.01 ± 1.57 15.43-32.76 | 24.16 ± 1.38 14.85-29.54 |
| Proboscis epimastigotes | Day 13 (23) | 18.45 ± 0.63 14.75-26.22 | 2.19 ± 0.07 1.54-2.92 | 6.80 ± 0.37 4.30-10.47 | -1.40 ± 0.12-2.57-0.00 | 5.44 ± 0.32 3.31-8.98 | 2.53 ± 0.08 1.93-3.26 | 1.23 ± 0.06 0.70-1.82 | 11.53 ± 0.48 7.64-16.08 | 13.02 ± 0.44 9.16-17.76 |
|  | Day 16 (26) | 25.65 ± 1.26 17.32-42.04 | 1.94 ± 0.07 1.21-2.58 | 10.96 ± 0.64 5.49-17.23 | -2.11 ± 0.18 -3.44--0.46 | 9.47 ± 0.58 4.84-17.08 | 2.63 ± 0.14 1.62-4.59 | 1.17 ± 0.06 0.70-2.03 | 13.71 ± 0.70 9.32-20.76 | 16.16 ± 0.82 10.57-24.96 |
|  | Day 19 (18) | 31.20 ± 2.16 20.03-59.52 | 1.93 ± 0.11 1.42-3.45 | 14.61 ± 1.70 6.26-35.81 | -2.71 ± 0.22-4.26--0.82 | 11.52 ± 1.41 5.36-32.85 | 3.06 ± 0.14 1.75-4.22 | 1.28 ± 0.07 0.74-2.00 | 17.87 ± 1.35 11.56-26.13 | 19.67 ± 1.18 14.30-28.58 |
|  | Day 24  (9) | 21.37 ± 1.13 14.68-25.05 | 2.58 ± 0.15 1.77-3.31 | 9.94 ± 0.90 6.96-14.16 | -2.00 ± 0.511-4.08--0.03 | 7.97 ± 0.74 3.52-10.74 | 2.22 ± 0.16 1.67-2.88 | 1.15 ± 0.13 0.57-1.79 | 11.04 ± 0.52 7.72-12.27 | 13.41 ± 0.60 11.16-16.40 |
|  | Day 28 (34) | 28.00 ± 1.10 17.47-40.70 | 2.00 ± 0.07 1.22-2.98 | 13.50 ± 1.12 3.18-30.71 | -2.11 ± 0.18 -4.30--024 | 11.39 ± 1.01 1.92-28.00 | 2.43 ± 0.11 1.07-3.52 | 1.10 ± 0.05 0.34-1.89 | 14.50 ± 0.68 7.14-22.85 | 16.61 ± 0.63 9.39-23.62 |
|  | Day 31 (25) | 27.45 ± 2.29 16.71-63.29 | 2.12 ± 0.09 1.31-2.87 | 13.90 ± 1.90 3.47-41.75 | -2.18 ± 0.26 -5.20-0.25 | 11.72 ± 1.75 2.87-39.34 | 2.32 ± 0.12 1.42-3.38 | 1.11 ± 0.07 0.41-2.01 | 13.54 ± 0.69 9.79-22.75 | 15.72 ± 0.76 11.23-25.02 |
|  | Day 77 (91) | 32.18 ± 1.15 13.39-75.23 | 2.27 ± 0.05 1.55-3.78 | 19.20 ± 1.20 7.73-53.30 | -3.52 ± 0.19 -9.43-0.02 | 14.67 ± 0.87 2.66-50.60 | 3.34 ± 0.07 1.62-5.36 | 1.37 ± 0.03 0.52-2.25 | 14.53 ± 0.55 7.70-25.34 | 17.50 ± 0.28 10.11-29.48 |
| Proboscis metacyclics | Day 24  (4) | 11.00 ± 0.28 10.19-11.51 | 1.93 ± 0.13 1.67-2.29 | 0.75 ± 0.23 0.29-1.39 | 2.72 ± 0.48 2.07-4.14 | 4.00 ± 0.30 3.56-4.89 | 1.77 ± 0.15 1.39-2.04 | 0.76 ± 0.13 0.57-1.14 | 10.25 ± 0.37 9.54-10.91 | 7.01 ± 0.34 6.31-7.82 |
|  | Day 77  (3) | 14.07 ± 1.07 12.49-16.11 | 1.46 ± 0.10 1.26-1.60 | 0.52 ± 0.11 0.41-0.75 | 3.54 ± 0.06 3.42-3.62 | 4.78 ± 0.35 4.19-5.39 | 2.80 ± 0.12 2.66-3.04 | 1.04 ± 0.06 0.93-1.13 | 13.54 ± 0.96 12.08-15.36 | 9.29 ± 0.86 7.74-10.72 |
| Cibarial trypomastigotes | Day 77 (14) | 35.90 ± 2.13 23.07-54.33 | 2.15 ±0.14 1.27-3.09 | 4.62 ± 0.84 0.08-12.54 | 2.73 ± 0.41 0.34-6.45 | 8.10 ± 0.73 4.09-15.54 | 3.77 ± 0.23 2.07-5.01 | 1.26 ± 0.09 0.92-1.87 | 31.28 ± 1.61 22.23-41.79 | 27.81 ± 1.63 14.83-38.79 |
| Cibarial epimastigotes | Day 77 (144) | 31.56 ± 0.79 10.92-56.56 | 2.46 ± 0.03 1.47-3.56 | 16.70 ± 0.71 2.37-40.53 | -2.99 ± 0.15 -8.92-0.04 | 13.42 ± 0.56 1.85-33.13 | 2.96 ± 0.05 1.42-5.12 | 1.42 ± 0.02 0.69-2.56 | 15.38 ± 0.39 8.55-35.83 | 18.16 ± 0.38 9.07-36.31 |
